# Ce=O Terminated CeO_2_

**DOI:** 10.1002/anie.202101771

**Published:** 2021-05-11

**Authors:** David C. Grinter, Michael Allan, Hyun Jin Yang, Agustín Salcedo, Gustavo E. Murgida, Bobbie‐Jean Shaw, Chi L. Pang, Hicham Idriss, M. Verónica Ganduglia‐Pirovano, Geoff Thornton

**Affiliations:** ^1^ Department of Chemistry and London Centre for Nanotechnology University College London 20 Gordon Street London WC1H 0AJ UK; ^2^ Diamond Light Source Diamond House, Harwell Science and Innovation Campus Didcot OX11 0DE UK; ^3^ Departamento de Ingeniería Química, Facultad de Ingeniería Universidad de Buenos Aires Ciudad Universitaria C1428EGA Buenos Aires Argentina; ^4^ Centro Atómico Constituyentes GIyA CNEA San Martín Consejo Nacional de Investigaciones Científicas y Tecnicas C1033AAJ Buenos Aires Argentina; ^5^ Surface Science and Advanced Characterisation Chemical Sciences Division SABIC-CRD at Kaust Thuwal 23955 Saudi Arabia; ^6^ Instituto de Catálisis y Petroleoquímica CSIS C/Marie Curie 2 28049 Madrid Spain

**Keywords:** cerium dioxide, density functional calculations, heterogeneous catalysis, multiple bonds, scanning tunnelling microscopy

## Abstract

Multiply bonded lanthanide oxo groups are rare in coordination compounds and have not previously been reported for a surface termination of a lanthanide oxide. Here we report the observation of a Ce=O terminated ceria surface in a CeO_2_(111)‐($\sqrt{3}$ ×$\sqrt{3}$ )R30° reconstruction of ≈3 nm thick ceria islands prepared on Pt(111). This is evidenced by scanning tunnelling microscopy (STM), low energy electron diffraction (LEED) and high‐resolution electron energy loss spectroscopy (HREELS) measurements in conjunction with density functional theory (DFT) calculations. A Ce=O stretching frequency of 775 cm^−1^ is observed in HREELS, compared with 766 cm^−1^ calculated by DFT. The calculations also predict that the Ce=O bond is weak, with an oxygen vacancy formation energy of 0.85 eV. This could play an important role in the facile removal of lattice oxygen from CeO_2_, accompanied by the reduction of Ce^IV^ to Ce^III^, which is a key attribute of ceria‐based systems in connection with their unique catalytic properties.

## Graphical abstract

A combined experimental and computational study of the ($\sqrt{3}$ ×$\sqrt{3}$ )R30° reconstruction of the CeO_2_(111) surface of an ultrathin ceria film is presented. The evidence collected indicates that the film is terminated by Ce=O groups.

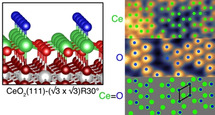

Ceria is a common component of many heterogeneous catalysts employed for important industrial processes including CO oxidation, CO_2_ hydrogenation, water–gas–shift, methane oxidation and methanol reforming.[^1^, ^2^, ^3^] Key to its catalytic function is the facile switching between Ce^III^ and Ce^IV^; the subsequent ease of oxygen vacancy formation leading to excellent oxygen storage capacity as well as influencing the adsorption of active metal nanoparticles.[^4^, ^5^] Moreover, oxygen adsorption and activation that results in the formation of peroxide (O_2_^2−^), superoxide (O_2_^−^), and weakly bound oxygen (O_2_^δ−^) species has been linked to the dynamic exchange of lattice oxygen.[^6^, ^7^, ^8^] A recent study of a CeO_2_‐Rh inverse model catalyst concluded that oxygen spill‐over from the metal also plays an important role in regeneration.^[9]^ Near‐surface oxygen vacancies are clearly important in oxygen exchange, with calculations indicating that subsurface oxygen vacancies are stabilized at the CeO_2_(111) surface.[^10^, ^11^, ^12^, ^13^] Here we report the observation of a Ce=O terminated (111) surface of ceria. Calculations suggest that this oxo species could play a crucial role in the mechanism of oxygen vacancy formation.

The lowest energy surface of CeO_2_ is the stoichiometric O‐terminated (111) surface with an ABC stacking of O‐Ce‐O trilayers.^[14]^ As with other reducible oxides, the surface reconstruction is dependent on the oxygen chemical potential^[15]^ (Supporting Information, Table S1). Here we focus on the CeO_2_(111) ($\sqrt{3}$ ×$\sqrt{3}$ )R30° reconstruction prepared as ultrathin islands on Pt(111). This reconstruction has been reported in an earlier study,^[16]^ although its precise origin and structure was not fully understood until now. The formation of this phase requires a slightly lower O_2_ partial pressure for the oxidation stage compared with that of the (1×1) bulk terminated surface (1×10^−7^ mbar vs. 5×10^−6^ mbar). This results in changes to the morphology of ceria islands as well as the surface periodicity. Islands with the ($\sqrt{3}$ ×$\sqrt{3}$ )R30° reconstructed surface, shown in Figure 1 a, are imaged with atomic resolution (Figure 1 b,c). The islands are significantly thicker than those observed for the (1×1) (Supporting information, Figure S1); 2.6–3 nm (9–10 O‐Ce‐O trilayers) as seen in Figure 1 d, compared with 1‐1.2 nm (3–4 O‐Ce‐O trilayers). Features in Figure 1 c have a periodicity of 0.59±0.03 nm and are rotated 30° with respect to the (1×1) lattice, which corresponds to a ($\sqrt{3}$ ×$\sqrt{3}$ )R30° termination. The ($\sqrt{3}$ ×$\sqrt{3}$ )R30° reconstruction is also evidenced in the Low Energy Electron Diffraction (LEED) pattern (Supporting Information, Figure S2). This was unstable after a few seconds of exposure to the LEED beam (few μA), leaving the (1×1) pattern.

**Figure 1:**
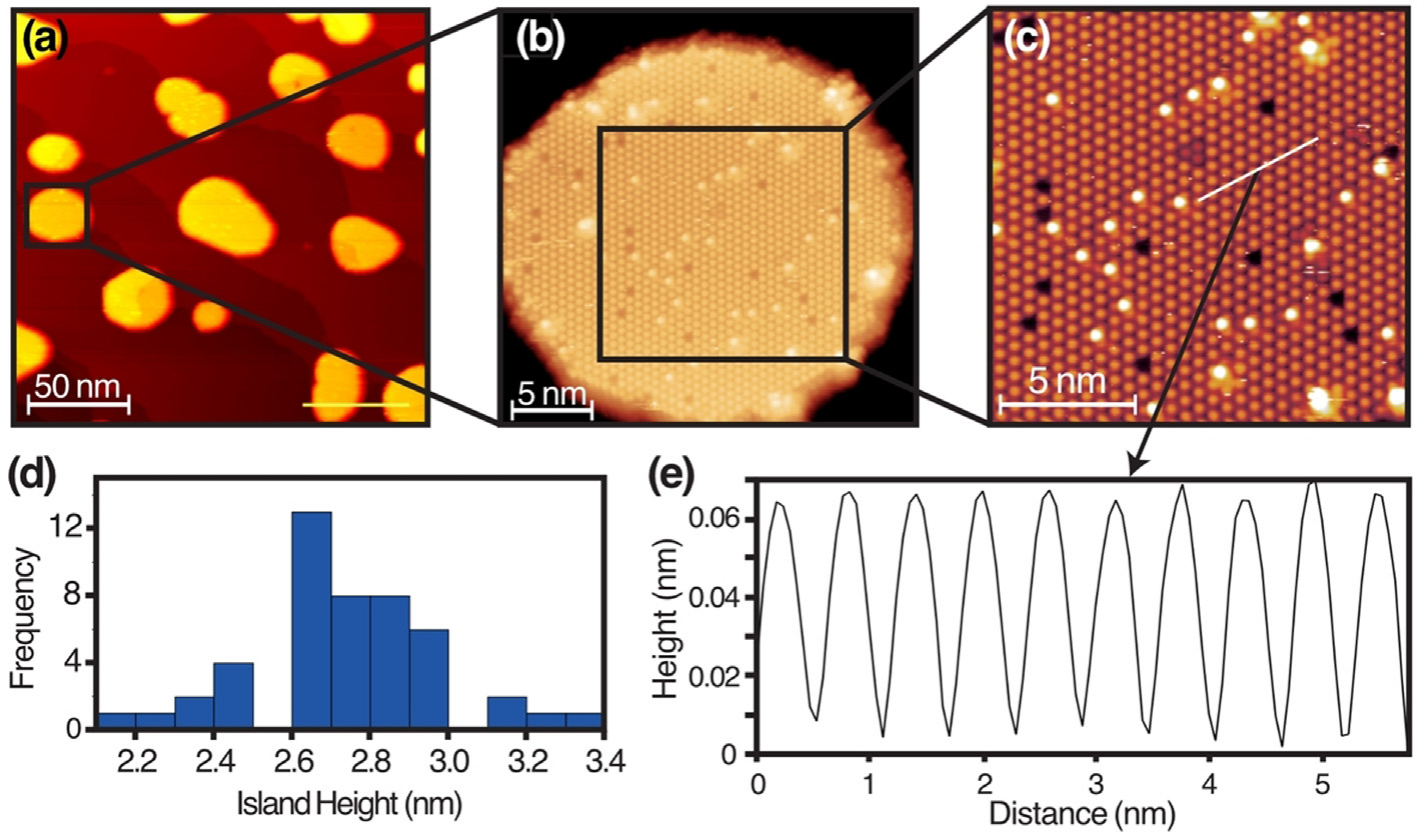
STM images of CeO_2_(111)‐($\sqrt{3}$ ×$\sqrt{3}$ )R30° islands. a) Large‐area STM image of ceria islands on Pt(111) (200×200 nm^2^). b) Filled‐states STM image of a ceria island (25×25 nm^2^), c) Magnified image of (b) of the atomically resolved reconstructed surface (15×15 nm^2^). d) Histogram of the average island heights in (a) with a bin width of 0.1 nm. e) Line profile corresponding to the white line in (c). (*V*_s_=−4.4 V, *I*_t_=10 pA.)

Point defects are also observed in the STM image in Figure 1 c, including vacancies as depressions, and adatoms or molecules as protrusions (likely water adsorbed from the residual vacuum). The enhanced corrugation (50 pm vs. 5 pm) of the ($\sqrt{3}$ ×$\sqrt{3}$ )R30° reconstructed surface compared with the (1×1) (Supporting Information, Figure S1) is evidenced in the line profile shown in Figure 1 e. This enhanced corrugation as well as the similarity to low temperature STM images of V=O on V_2_O_3_(001)^[17]^ suggest that the features in Figure 1 c correspond to Ce=O. Although multiply bonded lanthanide oxo species are rare, there are recent reports of Ce=O containing compounds.[^18^, ^19^] One is Ce=O(LOEt)_2_‐(H_2_O)]⋅MeC(O)NH_2_, where (LOEt)_2_ is a Kläui oxygen tripodal ligand.^[18]^ The Ce=O bond length is 1.86 Å, with a stretching frequency of 684 cm^−1^.^[18]^ There is a single report of a Ce=O oxo group that is not stabilized by H bonding or an alkali metal coordination, where the bond length is 1.84 Å.^[19]^ Further support for a Ce=O surface termination can be found in a comparison of filled and empty states STM images, shown in Figure 2. Filled (empty) state images sample valence (conduction) band states and hence are sensitive to O (Ce).[^5^, ^20^] Remarkably, the positions of the protrusions in the filled state images are a subset of those in the empty state images, indicating that O sits directly atop Ce (Figure 2 c).

**Figure 2:**
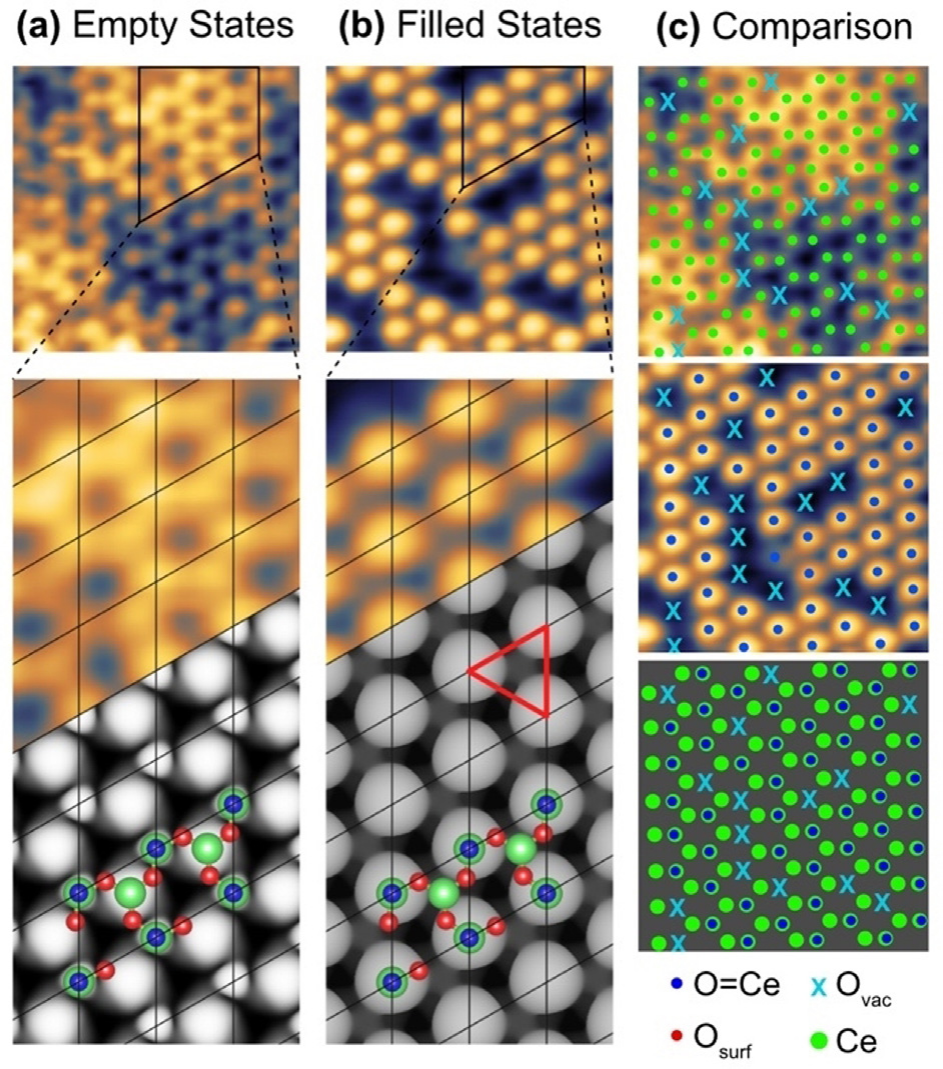
Experimental and DFT‐simulated STM images of the CeO_2_(111)‐($\sqrt{3}$ ×$\sqrt{3}$ )R30° reconstructed surface. a) Empty states; *V=*+1.7 V. b) Filled states; *V=*−4 V, recorded by dual mode imaging. The DFT calculations are based on the models shown, in which the larger spheres are Ce and the smaller are O. The Ce=O bond is formed by a green Ce and dark blue O. The red triangle denotes a threefold position where there is a Ce atom surrounded by Ce=O species. c) The right panel shows the superposition of the marked protrusions in the empty (Ce) and filled states (O and O_vac_) images.

We further test the validity of the Ce=O hypothesis through Density Functional Theory (DFT) calculations and High‐Resolution Electron Energy Loss (HREELS) measurements. The Ce=O terminated model of the ($\sqrt{3}$ ×$\sqrt{3}$ )R30° reconstruction, shown in Figure 3, was confirmed to be a local energy minimum by the absence of imaginary vibrational frequencies. A Ce‐O‐Ce bridge termination is also possible for the same Ce_2_O_4_ stoichiometry of the uppermost trilayer (denoted TL1), which is more stable by Δ*E*_Ce=O→bridge_=−0.94 eV (Supporting Information, Figure S3), although this structure has not been observed experimentally.

**Figure 3:**
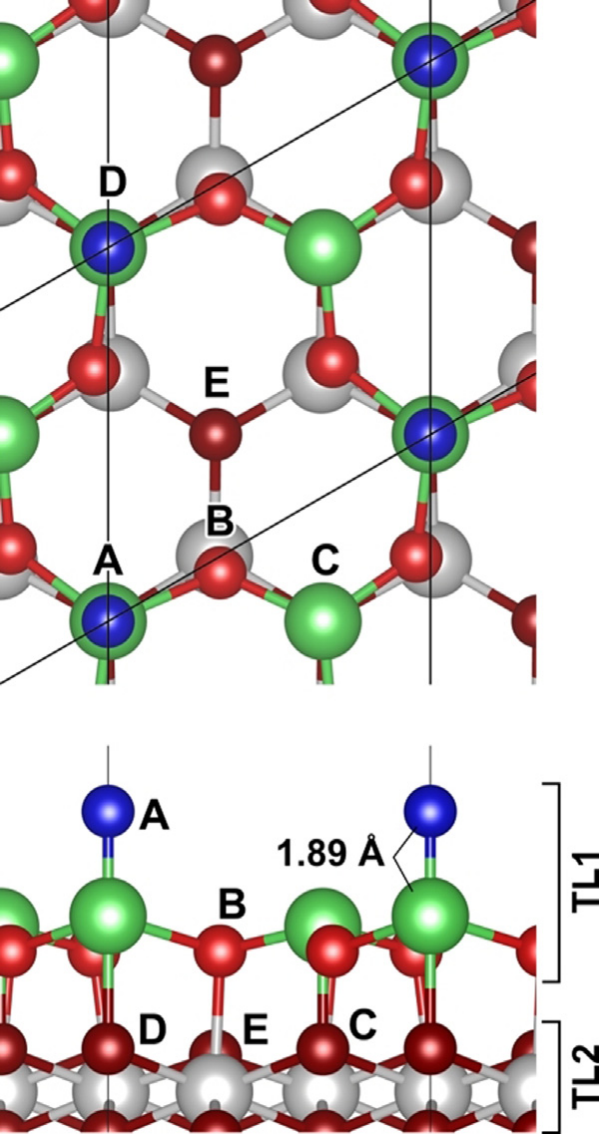
Top and side views of the proposed Ce=O termination of CeO_2_(111)‐($\sqrt{3}$ ×$\sqrt{3}$ )R30°. The larger spheres are Ce and the smaller are O. The Ce=O bond is between the green Ce to blue O, with a calculated bond distance of 1.89 Å. The inequivalent O atoms in the outermost three oxygen layers are denoted A (Ce=O in TL1), B (TL1) and C–E (TL2).

A likely explanation is that the Ce=O terminated phase is formed out of equilibrium, in other words it is a metastable structure formed from kinetically limited crystallization. Another example of this phenomenon is found in the case of TiO_2_(100)‐(1×3) reconstructions.^[21]^ In the simulated filled states STM image in Figure 2 b, the threefold positions surrounded by Ce=O species appear grey where there is a Ce atom, and black at the location of the Ce defect of the Ce=O reconstruction. In the simulated empty states image (Figure 2 a), bright spots appear at the location of the Ce=O species, matching the experimental data well.

Calculated IR spectra for the Ce=O‐terminated and unreconstructed CeO_2_(111)‐(1×1) surfaces are shown in Figure 4. The most intense bands for the CeO_2_(111)‐(1×1) surface appear at 538 and 526 cm^−1^, assigned to F_1u_‐type vibrations where the near‐surface O atoms move along the [111] direction (Fuchs–Kliewer modes). The band at 367 cm^−1^ is assigned to the transversal stretch of the outermost oxygen atoms.^[22]^ Fuchs–Kliewer modes are also observed for the Ce=O‐terminated ($\sqrt{3}$ ×$\sqrt{3}$ )R30° surface, at 516 and 529 cm^−1^. The Ce=O surface also shows a distinctive band at 766 cm^−1^, corresponding to the stretching vibration of the Ce=O double bond. For comparison, calculations for the O‐bridge‐($\sqrt{3}$ ×$\sqrt{3}$ )R30° reconstructed surface (Supporting Information, Figure S4) predict two similar F_1u_‐like modes, shifted to 537 and 527 cm^−1^. The transversal vibration modes of the bridge O and the subsurface O located below it in TL1 (Supporting Information, Figure S3, A and B, respectively) appear at 502 and 586 cm^−1^, respectively. The longitudinal stretching frequency of the bridging oxygen is 682 cm^−1^ (not IR‐active). Supporting Information, Table S2 contains a complete list of calculated frequencies.

**Figure 4:**
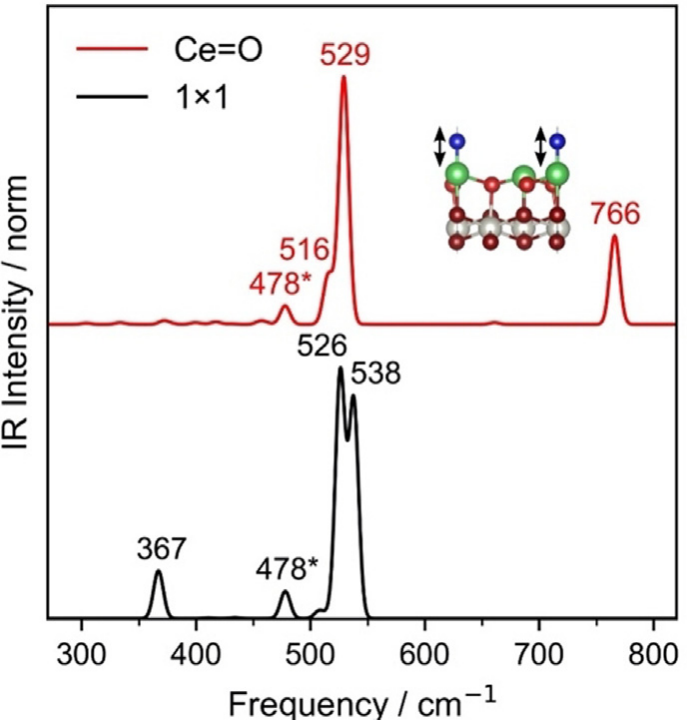
Calculated IR spectra for the Ce=O‐terminated ($\sqrt{3}$ ×$\sqrt{3}$ )R30°‐reconstructed and (1×1) unreconstructed CeO_2_(111) surfaces. The model for the Ce=O‐terminated ($\sqrt{3}$ ×$\sqrt{3}$ )R30° surface is the same as that in Figure 3. The 478 cm^−1^ mode observed for both is ascribed to vibrations against the fixed layer of the DFT slab.^[22]^

HREEL spectra of the ceria islands are shown in Figure 5. The Ce=O stretch is expected at 680–780 cm^−1^ from previous studies of cerium coordination compounds,[^18^, ^23^] with H‐bonding thought to give rise to the red shifted end of this spectrum. Our predicted frequency for surface Ce=O (766 cm^−1^) is in line with these values. Measurements were recorded from a fresh region of the sample, corresponding to the CeO_2_(111)‐($\sqrt{3}$ ×$\sqrt{3}$ )R30° reconstruction (Figure 5 a), and for comparison from an area exposed to the LEED beam where a (1×1) pattern was observed (Figure 5 b). The HREEL spectrum of the (1×1) surface in Figure 5 b shows significant loss features at 571, 1057 and 2107 cm^−1^. The loss at 571 cm^−1^ corresponds to the surface optical phonon mode of CeO_2_(111)‐(1×1).^[24]^ The loss at 2107 cm^−1^ is assigned to the CO‐Pt(111) atop stretching frequency as seen in a control experiment for CO/Pt(111) (Supporting Information, Figure S5). The losses centered at 1057 cm^−1^ are also seen in earlier data from CeO_2_(111)‐(1×1)/Pt(111),^[24]^ being a combination of multiple loss peaks for the surface optical phonon and features associated with Pt_x_Ce alloy on the surface. The component peaks are better resolved in the spectrum of the ($\sqrt{3}$ ×$\sqrt{3}$ )R30° reconstruction and are identified at 536 (ν_1_), 775 (ν_2_), 1014 (ν_3_), 1144 (ν_4_), 1247 (ν_5_) and 2081 (ν_6_) cm^−1^. The only significant difference between the spectra in Figure 5 a,b is the appearance of a loss at 775 cm^−1^ for the reconstructed surface, which is very close to the calculated value for the Ce=O stretch frequency. As a further comparison, an HREEL spectrum from an un‐reconstructed CeO_2_(111)(1×1) film (prepared at higher pO_2_) is displayed in Figure 5 c; this matches qualitatively with that of Figure 5 b and displays no sign of the Ce=O stretch. Small differences in the peak positions are likely due to variations in the film morphology and Pt support. Due to the higher oxygen pressure during preparation of the film in Figure 5 c we would expect a smaller amount of residual PtCe surface alloy to be present, explaining the lower intensity of the peak at ≈1100 cm^−1^.

**Figure 5:**
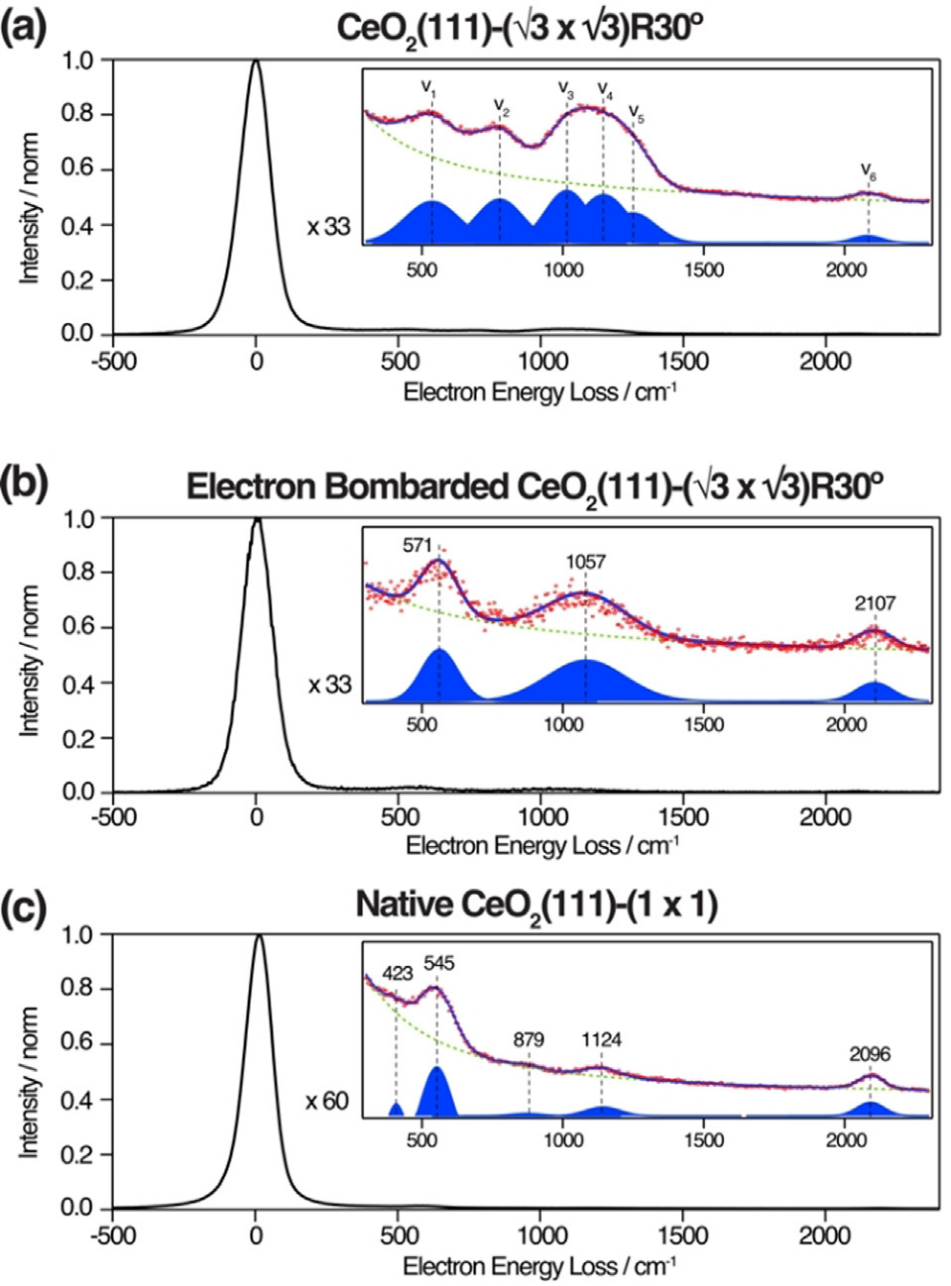
HREEL spectra of CeO_2_(111): a) area of the sample with the ($\sqrt{3}$ ×$\sqrt{3}$ )R30° surface reconstruction. b) A (1×1) area of the same sample following irradiation by the LEED electron beam. c) A native CeO_2_(111) film with (1×1) termination. Inset: magnified views of the 300–2300 cm^−1^ regions.

This spectroscopic evidence validates the conclusion from STM and DFT that there is a Ce=O termination of CeO_2_(111). Ce=O bonds are expected to be weak based simply on their rarity in coordination compounds. This suggests the possibility that they could act as active sites for the formation of oxygen vacancies. To test this hypothesis, oxygen vacancy formation energies were calculated (Supporting Information, Table S3). For the Ce=O‐terminated surface, we investigated the formation of oxygen vacancies in the upper three oxygen layers. We considered the five inequivalent types of oxygen atoms, labelled A–E in Figure 3. With ($\sqrt{3}$ ×$\sqrt{3}$ )R30° periodicity, a single oxygen vacancy corresponds to a vacancy concentration of *Θ*=0.33. *Θ* is defined as the number of oxygen vacancies divided by the total number of atoms in a non‐reduced and unreconstructed oxygen layer of the same cell (i.e. 3 atoms for ($\sqrt{3}$ ×$\sqrt{3}$ )R30° periodicity). We also investigated the same concentration of surface (s) and subsurface (ss) oxygen vacancies at the unreconstructed CeO_2_(111) surface and a bridge‐oxygen vacancy in the O‐bridge‐($\sqrt{3}$ ×$\sqrt{3}$ )R30° reconstructed surface. At the unreconstructed CeO_2_(111) surface with *Θ*=0.33, the most stable vacancy is subsurface, with one Ce^3+^ ion in TL1 and one in TL2 (*E*_vac_=2.05 eV). This value is in good agreement with previous work as it lies in between the *E*_vac_ values calculated for subsurface vacancy concentrations of 0.25 and 0.50 (1.91 and 2.31 eV, respectively).^[11]^ Removal of the terminating oxygen from the O‐bridge‐($\sqrt{3}$ ×$\sqrt{3}$ )R30° reconstruction requires 1.81–2.02 eV, depending on the location of the excess charge. Vacancy formation on the Ce=O‐terminated surface is heavily favored, with energies of ≈1 eV. The most stable vacancy is subsurface V^E^ (cf. Figure 3, *E*_vac_=0.83 eV), but oxygen abstraction from the Ce=O species (V^A^) is also favorable, with virtually the same formation energy (*E*_vac_=0.85 eV). The filled states STM image in Figure 2 b reveals several oxygen vacancies in the surface layer, imaged as dark holes. At the corresponding positions in the empty states image (highlighted with “X” in Figure 2 c) there is an absence of Ce^4+^ features that are observed elsewhere on the surface. This can be understood on the basis of previous work on reduced CeO_2_(111)^[20]^ in which it was concluded that while Ce^4+^ gave rise to bright features, Ce^3+^ ions did not. The presence of Ce^3+^ below the O vacancies on the ($\sqrt{3}$ ×$\sqrt{3}$ )R30° reconstruction is consistent with the most stable excess charge distribution obtained upon oxygen abstraction from the Ce=O species (Supporting Information, Table S3, Figure S6). Moreover, neither Ce^3+^ below the vacancy in TL1 nor that in TL2 appear as a bright feature in the calculated empty states image. Furthermore, missing or attenuated features in the empty state image at Ce positions that are not located just below missing O sites, such as the large dark patches in Figure 2 a and Figure S7, also correspond to Ce^3+^ ions in TL1 and are likely to result from deeper lying subsurface oxygen vacancies. The formation energy of subsurface oxygen vacancies, with one excess electron in TL1 and the other one in TL2, lie within a range of 0.85–1.08 eV (Table S3). The proposed structure for the Ce=O‐terminated CeO_2_(111)‐($\sqrt{3}$ ×$\sqrt{3}$ )R30° reconstruction, as well as the calculated low energy vacancy structures, allow us to satisfactorily explain the experimentally observed filled and empty state STM images.

In summary, a CeO_2_(111)‐($\sqrt{3}$ ×$\sqrt{3}$ )R30° reconstruction of an ultrathin ceria film prepared on Pt(111) has been studied using surface imaging (STM), diffraction (LEED), and vibrational spectroscopy (HREELS). The results all point to the formation of a Ce=O termination, representing the first observation of a surface multiply bonded lanthanide oxo species. This interpretation is validated by DFT calculations of the filled and empty states STM images for the Ce=O model, which closely match the experimental images. Moreover, a Ce=O stretch frequency of 766 cm^−1^ is predicted, which is in excellent agreement with the HREELS experimental value of 775 cm^−1^. The calculations also predict that Ce=O species on CeO_2_(111) could act as an active site for the formation of oxygen vacancies, which play a key role in ceria‐based heterogeneous catalysis.

## Conflict of interest

The authors declare no conflict of interest.

## Supporting information

As a service to our authors and readers, this journal provides supporting information supplied by the authors. Such materials are peer reviewed and may be re‐organized for online delivery, but are not copy‐edited or typeset. Technical support issues arising from supporting information (other than missing files) should be addressed to the authors.

Supplementary

Supplementary

Supplementary

Supplementary

Supplementary

Supplementary

Supplementary

Supplementary

Supplementary

Supplementary

Supplementary
