# Supplementary material for: Ce=O Terminated CeO2
Source: Angew Chem Int Ed Engl. 2021 May 11;60(25):13835–9. doi: 10.1002/anie.202101771 (PMC8251574; doi:10.1002/anie.202101771)
Supplement: Supplementary file 8 — Supplementary [file ANIE-60-13835-s008.pdf]

Angewandte Chemie
Eine Zeitschrift der Gesellschaft Deutscher Chemiker

Supporting Information

Ce=O Terminated CeO_2

David C. Grinter, Michael Allan, Hyun Jin Yang, Agustín Salcedo, Gustavo E. Murgida, Bobbie-Jean Shaw, Chi L. Pang, Hicham Idriss, M. Verónica Ganduglia-Pirovano, and Geoff Thornton**

anie_202101771_sm_miscellaneous_information.pdf
anie_202101771_sm_bridge_502cm.mp4
anie_202101771_sm_bridge_527cm.mp4
anie_202101771_sm_bridge_537cm.mp4
anie_202101771_sm_bridge_586cm.mp4
anie_202101771_sm_Ce=O_516cm.mp4
anie_202101771_sm_Ce=O_529cm.mp4
anie_202101771_sm_Ce=O_766cm.mp4
anie_202101771_sm_unreconstr_367cm.mp4
anie_202101771_sm_unreconstr_526cm.mp4
anie_202101771_sm_unreconstr_538cm.mp4

Contents

Experimental Methods
Computational Methods
Table S1: Comparison of ceria surface reconstructions
Table S2: Calculated vibrational frequencies and IR intensities
Table S3: Oxygen vacancy formation energies
Figure S1: STM of $CeO_2(111)$-(1 × 1)
Figure S2: LEED of $CeO_x(111)$-(√3 × √3)R30°
Figure S3: O-bridge-(√3 × √3)R30° reconstruction
Figure S4: Simulated IR spectra of the O-bridge-(√3 × √3)R30° reconstruction
Figure S5: HREELS spectrum of CO/Pt(111)
Figure S6: Simulated STM image of the reduced Ce=O-(√3 × √3)R30° reconstruction
Figure S7: Dual mode atomically resolved STM

Experimental Methods

The Pt(111) substrate was prepared by successive cycles of Ar^+ sputtering (1.5 keV) and annealing at 1100 K in UHV. Sample cleanliness was confirmed by observing a well-ordered Low Energy Electron Diffraction (LEED) pattern for Pt(111)-(1 × 1), with no impurities detected using Auger Electron Spectroscopy (AES). In order to facilitate the removal of carbon and embedded impurities, occasional cycles of annealing were performed at ~1000 K in 1 x 10^{-7} mbar O_2. The ceria islands were prepared using a post-oxidative process by first evaporating cerium metal (Alfa Aesar, 99.9%) via physical vapour deposition onto the clean Pt(111) substrate at 300 K from an electron-bombardment type evaporator. This was subsequently annealed at 1000 K for 20 minutes in a partial pressure of up to 5 x 10^{-6} mbar O_2 for the (1 × 1) terminated islands, and up to 1 x 10^{-7} mbar O_2 for the ($\sqrt{3} \times \sqrt{3}$)R30° islands. The samples were then allowed to cool for 10 to 20 minutes in these respective partial pressures of O_2. The temperatures were monitored using a Minolta infrared pyrometer alongside a type K thermocouple. Scanning Tunnelling Microscopy (STM) measurements were performed using an Omicron LT-STM with a base pressure of 1 x 10^{-11} mbar operated at 78 K using chemically-etched tungsten tips. LEED and X-ray photoelectron spectroscopy (XPS) were used to check the periodicity and chemical composition of the surface, respectively.

The HREELS experiments were carried out using a VSW HREELS system housed in a UHV chamber with a base pressure of 3 x 10^{-10} mbar at room temperature. This instrument is also equipped with LEED/AES. HREELS measurements were carried out in the specular geometry at 45° off-normal. An incident electron energy of 10 eV was used during HREELS measurements, with the FWHM of the elastic peak being about 15 meV. Each spectrum was collected at 300 K, with a dwell time of 0.1 s, step size of 0.5 meV and were produced by averaging a minimum of 50 individual scans. To ensure no degradation of the film was

occurring, a single scan was collected before collecting the averaged scans to allow comparison. Spectra are normalized to the elastic peak.

In this work, one ceria monolayer (ML) is defined as one O–Ce–O trilayer unit with a thickness of 0.31 nm. As the ceria islands exhibit a Volmer-Weber growth, the total film thickness is presented in monolayer equivalent (MLE), where the coverage is estimated from the island thicknesses and the surface area fraction measured by STM.

Computational Methods

Spin-polarized DFT calculations were performed using the slab-supercell approach, with the Vienna Ab-initio Simulation Program (VASP, version 5.4.4)[1]. Ce (4f, 5s, 5p, 5d, 6s) and O (2s, 2p), electrons were treated explicitly as valence states within the projector augmented wave (PAW) method[2] with a plane-wave cut-off energy of 400 eV. Energies were calculated using the DFT+U approach by Dudarev et al.[3] ($U_{\mathrm{eff}} = U - J = 4.5$ eV for the Ce 4f electrons[4,5]) with the GGA-type exchange-correlation functional proposed by Perdew, Burke, and Ernzerhof (PBE)[6].

The Ce=O- and O-bridge-terminated ($\sqrt{3} \times \sqrt{3}$)R30°-reconstructed and (1 × 1)-unreconstructed CeO_2(111) surfaces were built using the calculated CeO_2 bulk equilibrium lattice constant (5.485 Å)[7]. For the calculation of vacancy formation energies and vibrational frequencies, we employed slabs containing 18 atomic layers (six O–Ce–O trilayers), whereas thinner slabs containing 9 atomic layers (three trilayers) were used to produce the simulated STM images. In both cases the surface unit cell parameters and the atomic positions of the bottom trilayer (TL) were kept fixed during geometry optimization, while the rest of the atoms were allowed to fully relax. The vacuum separation between consecutive slabs was ∼14 Å. During geometry optimization, total energies and forces were converged to at least 10^{-6} eV and 0.01 eV/Å respectively. Vibrational frequencies for the unreconstructed surface were

calculated with a (1×1) periodicity, employing a Γ-centred (8×8×1) k-point mesh. The remainder of the calculations were performed with a (√3 × √3)R30° periodicity employing a Γ-centred (4×4×1) k-point mesh, using the Monkhorst−Pack method[8]. Vibrational frequencies and normal modes were obtained by diagonalization of the Hessian matrix, calculated from finite differences with atomic displacements of ±0.015 Å. Infrared (IR) intensities for each normal mode were calculated as the square of the first derivative of the *z* component of the dynamic dipole moment. The computed spectra were smoothed by folding with a Gaussian with a half-width of 10 cm^{-1}. STM images were simulated within the Tersoff-Hamann approximation,[9] where a surface of constant current in an STM experiment can be identified with a surface of constant partial charge density in calculations. For the filled states images, states within an energy range of −0.7 eV to the Fermi level were considered, whereas for the empty states, the considered states were up to 1.6 eV above the Fermi level. In the simulated empty states STM images of the reduced Ce=O-(√3 × √3)R30° reconstruction (Figure S7), in order to improve the contrast, the occupied 4f states (Ce^{3+}) were treated explicitly as core states within the PAW method.

Surface Termination	Sample Morphology	References
CeO_2(111)-(1 × 1)	Single crystal as well as thick and thin films.	10–13
Ce_4O_7(111)-(2 × 2)	Thick and thin films.	13–15
Ce_7O_{12}(111)-(√7 × √7)R19.1°	Thick and thin films.	10,11,16,17
Ce_3O_5(111)-(√7 × √3)R19.1°	Thick film	11
Ce_3O_5(111)-(3 × 3)	Thick and thin films.	10,18,19
Ce_3O_5(111)-(√3 × √3)R30°	Thick and thin films.	11, 20
Ce_2O_3(111)-(4 × 4)	Single crystal as well as thick and thin films.	10,18,21

Table S1. Summary of reported terminations of CeO_2(111).

In the work by Olbrich et al.,[11] a (111)-oriented thick ceria film was annealed in ultrahigh vacuum at temperatures that were increased stepwise between 1020 and 1080 K. During this treatment, the surface of the thick ceria film passed through a series of atomically well-ordered vacancy structures with different stoichiometry, where the positions of the cations did *not* significantly change with respect to those of the CeO_2(111) surface; this is related to the higher mobility of O anions in ceria, as compared to Ce cations. In fact, the well-known cubic reduced Ce_xO_y bulk stable phases, namely, $Ce_{11}O_{20}$, Ce_7O_{12}, and Ce_2O_3-C type, keep the ceria cation sublattice almost intact. One of these reduced ceria surface structures has a (√3 × √3)R30º periodicity, therefore a structural model was created so as to match the experimental observations with respect to the periodicity of the vacancy structure and the fact that no major restructuring of the cation positions was observed. The so-obtained (√3 × √3)R30º structure was related to the Ce_3O_5 stoichiometry.

The Ce=O-terminated CeO_2(111) (√3 × √3)R30º reconstruction discussed in this work has been very differently obtained, namely, during the film preparation at low oxygen pressures. This surface differs from the reduced bulk-truncated CeO_2(111) with the same (√3 × √3)R30º periodicity previously discussed in that, in the Ce=O-terminated CeO_2(111) structure, a *Ce atom would be missing* in the outermost cationic plane when compared with the

hexagonal array of Ce atoms on CeO_2(111) (1 × 1) (see Figure 2, empty states). The proposed structural model for the Ce=O-terminated CeO_2(111) (√3 × √3)R30º reconstruction, with CeO_2 stoichiometry and a Ce vacancy in the outermost cationic plane (Figure 3), matches well the experimental observations through STM (see Figure 2, empty states) and HREELS measurements.

CeO_2(111)-(1 × 1)			Ce=O-(√3 × √3)R30°			O-bridge-(√3 × √3)R30°		
**Mode**	**freq (cm^{-1})**	**IR intensity**	**Mode**	**freq (cm^{-1})**	**IR intensity**	**Mode**	**freq (cm^{-1})**	**IR intensity**
**1**	**538**	**0.88562**	**1**	**766**	**0.36173**	1	682	0.00184
**2**	**526**	**1.00000**	2	661	0.00704	**2**	**586**	**0.35500**
3	517	0.01823	3	552	0.00000	3	560	0.00985
4	508	0.03262	4	552	0.00000	4	549	0.00082
**5**	**478**	**0.11087**	5	546	0.00072	5	548	0.00024
6	434	0.00000	6	546	0.00000	6	546	0.00200
7	434	0.00000	7	546	0.00000	7	545	0.00020
8	434	0.00340	8	546	0.00009	**8**	**537**	**0.98047**
9	426	0.00000	**9**	**529**	**1.00000**	**9**	**527**	**1.00000**
10	426	0.00000	10	526	0.00000	10	526	0.03208
11	424	0.00033	11	526	0.00002	11	526	0.01224
12	411	0.00002	12	522	0.04585	**12**	**522**	**0.16195**
13	411	0.00018	**13**	**516**	**0.18821**	**13**	**511**	**0.07124**
14	411	0.00245	14	510	0.00269	**14**	**502**	**0.43528**
15	402	0.00000	15	479	0.00000	**15**	**478**	**0.16653**
16	392	0.00000	16	479	0.00000	16	464	0.03381
17	391	0.00000	**17**	**478**	**0.07550**	17	460	0.00174
18	370	0.00002	18	461	0.00506	18	459	0.00219
19	370	0.00001	19	459	0.00000	19	458	0.00001
**20**	**367**	**0.19449**	20	459	0.00000	20	456	0.00130
21	328	0.00000	21	456	0.01198	21	451	0.00614
22	328	0.00000	22	454	0.00000	22	444	0.00251
23	307	0.00000	23	454	0.00000	23	437	0.00027
24	307	0.00000	24	434	0.00000	24	432	0.00009
25	287	0.00000	25	434	0.00000	25	431	0.00003
26	287	0.00000	26	431	0.00237	26	430	0.00005
27	273	0.00000	27	424	0.00000	27	429	0.00113
28	273	0.00000	28	424	0.00000	28	420	0.00312
29	225	0.00000	29	419	0.00491	29	417	0.00038
30	225	0.00000	30	417	0.00000	30	416	0.00059
31	196	0.00043	31	417	0.00000	31	415	0.00005
32	175	0.00250	32	417	0.00092	32	415	0.00033
33	139	0.00000	33	416	0.00446	33	408	0.00000
34	110	0.00000	34	404	0.00187	34	404	0.00020
35	110	0.00000	35	404	0.00000	35	402	0.00008
36	100	0.00000	36	403	0.00000	36	399	0.00001
37	100	0.00000	37	399	0.00608	37	396	0.00001
38	88	0.00060	38	394	0.00000	38	392	0.00027
39	82	0.00000	39	394	0.00000	39	386	0.00231
40	82	0.00000	40	388	0.00000	40	382	0.00244
41	55	0.00000	41	388	0.00000	41	381	0.00017
42	54	0.00000	42	383	0.00000	42	375	0.00034

43	30	0.00002	43	383	0.00000	43	372	0.00000
44	19	0.00000	44	379	0.00312	44	369	0.00049
45	19	0.00000	45	372	0.01457	45	364	0.00044
			46	352	0.00000	46	353	0.00036
			47	352	0.00000	47	343	0.00022
			48	344	0.00000	48	341	0.00004
			49	344	0.00000	49	339	0.00388
			50	343	0.00072	50	332	0.00005
			51	333	0.00695	51	330	0.00018
			52	331	0.00000	52	325	0.00172
			53	331	0.00000	53	323	0.00004
			54	324	0.00000	54	323	0.00158
			55	324	0.00000	55	314	0.00650
			56	307	0.00000	56	307	0.00585
			57	306	0.00000	57	304	0.00111
			58	304	0.00491	58	303	0.00056
			59	303	0.00000	59	300	0.00015
			60	303	0.00000	60	297	0.00116
			61	296	0.00019	61	294	0.00083
			62	287	0.00000	62	291	0.00068
			63	287	0.00000	63	284	0.00000
			64	284	0.00000	64	281	0.00002
			65	284	0.00000	65	278	0.00088
			66	272	0.00000	66	277	0.00008
			67	272	0.00000	67	270	0.00128
			68	263	0.00116	68	266	0.00104
			69	261	0.00000	69	263	0.00176
			70	261	0.00000	70	258	0.00200
			71	249	0.00022	71	253	0.00157
			72	244	0.00000	72	247	0.00096
			73	244	0.00000	73	238	0.00354
			74	233	0.00262	74	234	0.00116
			75	218	0.00000	75	228	0.00372
			76	218	0.00000	76	219	0.00109
			77	218	0.00038	77	217	0.00090
			78	214	0.00252	78	216	0.00422
			79	214	0.00000	79	214	0.00891
			80	213	0.00001	80	211	0.00100
			81	196	0.00015	81	204	0.00305
			82	191	0.00000	82	198	0.00820
			83	191	0.00000	83	188	0.00846
			84	187	0.00018	84	186	0.00069
			85	185	0.00000	85	185	0.00021
			86	185	0.00000	86	185	0.00008

87	183	0.00000	87	184	0.00083
88	183	0.00000	88	183	0.00313
89	183	0.00075	89	182	0.00135
90	181	0.00001	90	181	0.00040
91	180	0.00000	91	181	0.00103
92	180	0.00000	92	179	0.00004
93	179	0.00039	93	179	0.00001
94	179	0.00003	94	178	0.00010
95	177	0.00000	95	176	0.00035
96	177	0.00000	96	175	0.00389
97	176	0.00006	97	175	0.00748
98	170	0.00000	98	172	0.00013
99	170	0.00000	99	168	0.00011
100	160	0.00005	100	165	0.00879
101	150	0.00000	101	159	0.00179
102	150	0.00000	102	155	0.00696
103	138	0.00000	103	143	0.01876
104	138	0.00000	104	138	0.00000
105	126	0.00008	105	137	0.00009
106	124	0.00000	106	128	0.00639
107	124	0.00000	107	124	0.00008
108	123	0.00001	108	124	0.00027
109	123	0.00000	109	123	0.00058
110	118	0.00000	110	120	0.00069
111	118	0.00000	111	112	0.00609
112	109	0.00000	112	109	0.00209
113	109	0.00000	113	108	0.00333
114	103	0.00337	114	104	0.00759
115	99	0.00000	115	102	0.00003
116	99	0.00000	116	95	0.00021
117	87	0.00099	117	95	0.00002
118	83	0.00000	118	79	0.00116
119	83	0.00000	119	77	0.00205
120	57	0.00000	120	68	0.01371
121	57	0.00000	121	61	0.00260
122	32	0.00001	122	52	0.00267
123	21	0.00000	123	44	0.00073
124	20	0.00000	124	31	0.00031
125	14	0.00000	125	20	0.00001
126	10	0.00000	126	20	0.00001

Table S2. Calculated vibrational frequencies and their corresponding normalized IR intensity for the investigated surfaces. The main IR-active modes are indicated in bold.

**Vacancy**	$\boldsymbol{E}_{\mathbf{vac}}$	**Vacancy**	$\boldsymbol{E}_{\mathbf{vac}}$
**CeO_2(111)**			
V^{s}_{11}	2.17	V^{ss}_{11}	2.17
V^{s}_{12}	2.23	V^{ss}_{12}	2.05
V^{s}_{22}	2.48	V^{ss}_{22}	2.18
**Ce=O-(√3 × √3)R30°**			
V^{A}_{11}	1.37	V^{D}_{11}	1.44
$\mathbf{V^{A}_{12}}$	**0.85**	V^{D}_{12}	0.98
V^{A}_{22}	0.87	V^{D}_{22}	1.07
V^{B}_{11}	1.27	V^{E}_{11}	1.59
V^{B}_{12}	0.85	V^{E}_{12}	1.08
V^{B}_{22}	0.87	V^{E}_{22}	0.83
V^{C}_{11}	1.44		
V^{C}_{12}	1.01		
V^{C}_{22}	1.07		
**O-bridge-(√3 × √3)R30°**			
	V^{A}_{11}	2.20	
	V^{A}_{12}	1.81	
	V^{A}_{22}	1.83	

Table S3. Vacancy formation energy (E_{vac} w.r.t. ½ O_2) for the investigated structures (in eV) and a vacancy concentration of Θ=0.33. V^{x}_{nm} denotes a vacancy at the lattice O site *x* (see Figure 3 in the main text and Figure S7), and the sub-indices *n* and *m* indicate the trilayer in which the two excess electrons (Ce^{3+}) are located (1: TL1, 2:TL2). At Θ=0.33, excess electrons localize at Ce ions that are adjacent to the O vacancy. Θ is defined as the number of oxygen vacancies divided by the total number of atoms in a non-reduced and unreconstructed oxygen layer of the same cell (i.e. 3 atoms for (√3 × √3)R30° periodicity).

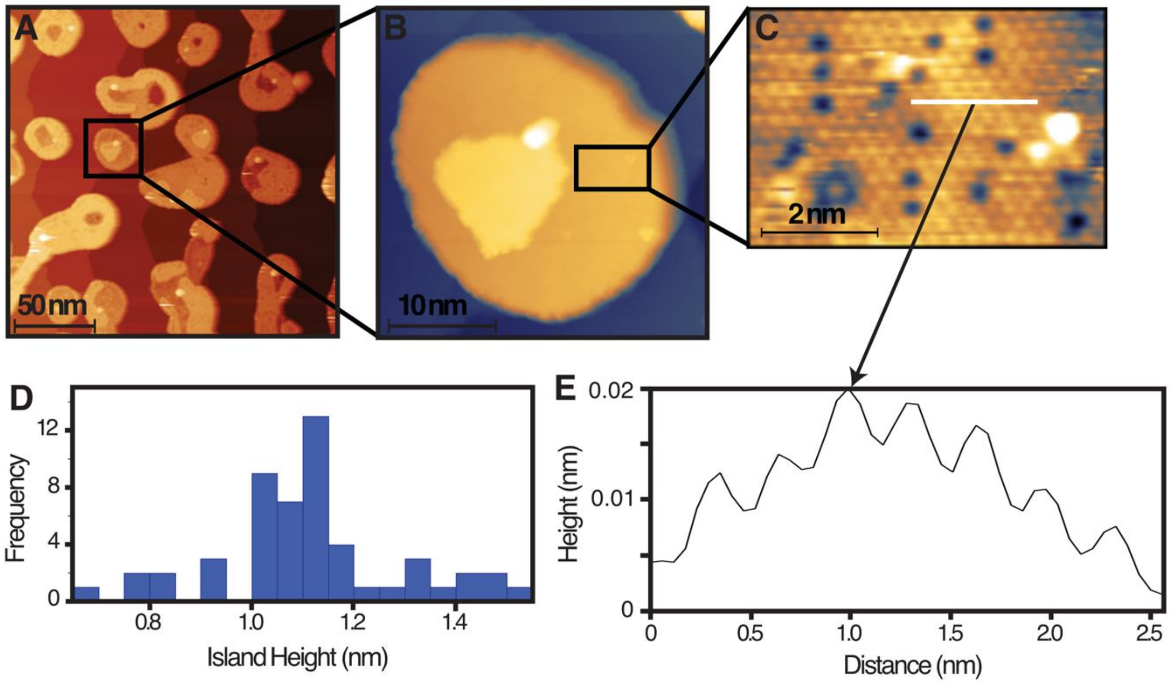

Figure S1. STM images of ultrathin CeO_2(111)-(1 × 1) islands prepared by post oxidation of Pt_xCe surface alloys. (a) A large area filled-states STM image of ceria islands grown on a Pt(111) substrate (200 × 200 nm^2, V_s = –2.5 V, 20 pA). (b) A representative filled-states STM image of an atomically resolved island (30 × 30 nm^2, V_s = –4.4 V, I_t = 20 pA). (c) Zoomed-in STM image of (b) showing the atomically resolved surface (4 × 6 nm^2, V_s = –4.4 V, I_t = 20 pA). (d) Histogram taken of the average island heights across image (a) with a bin width of 0.05 nm. (e) Line profile indicating the inter-atomic distance between the terminating oxygen atoms corresponding to the white line in (c).

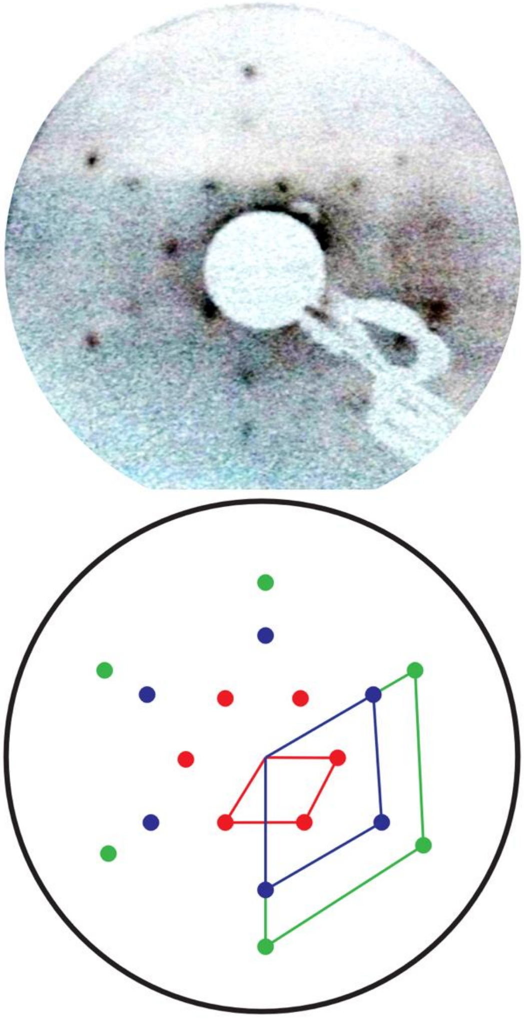

Figure S2. LEED pattern of ceria ultrathin islands prepared on a Pt(111) substrate under partially-oxidizing conditions.

Green: Pt(111)-(1 × 1)

Blue: CeO_2(111)-(1.37 × 1.37) [relative to Pt(111)-(1 × 1)]

Red: CeO_2(111)-(√3 × √3)R30° [relative to CeO_2(111)-(1 x 1)]

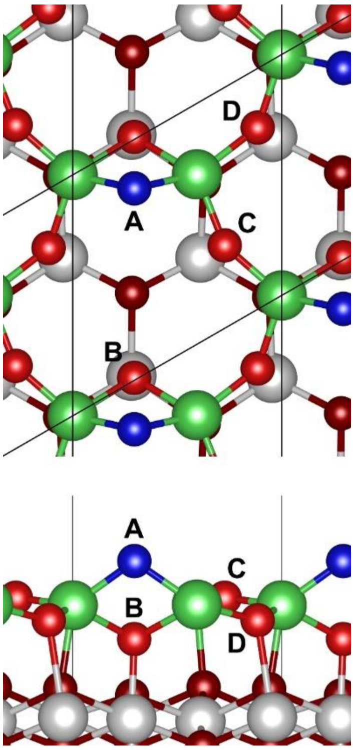

Figure S3. Top and side views of the calculated Ce–O–Ce bridge termination of the (√3 × √3)R30°-reconstructed $CeO_2(111)$ surface. The larger spheres are Ce and the smaller are O. The bridge oxygen is shown in blue. The inequivalent O atoms in TL1 are denoted A–D.

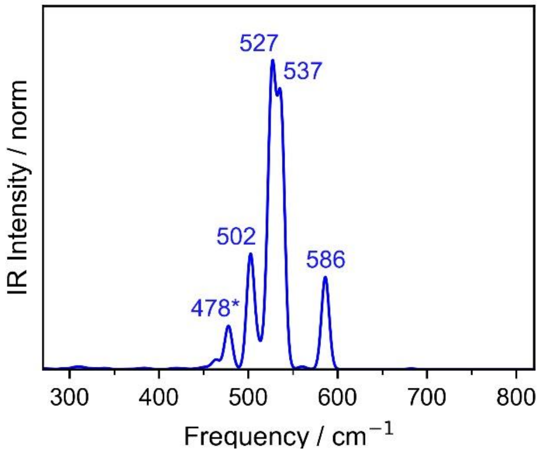

Figure S4. Calculated IR spectra for the O-bridge-(√3 × √3)R30°-reconstructed surface. The mode a 478 cm^{-1} observed for both slabs is ascribed to vibrations against the fixed layer of the DFT slab.[22]

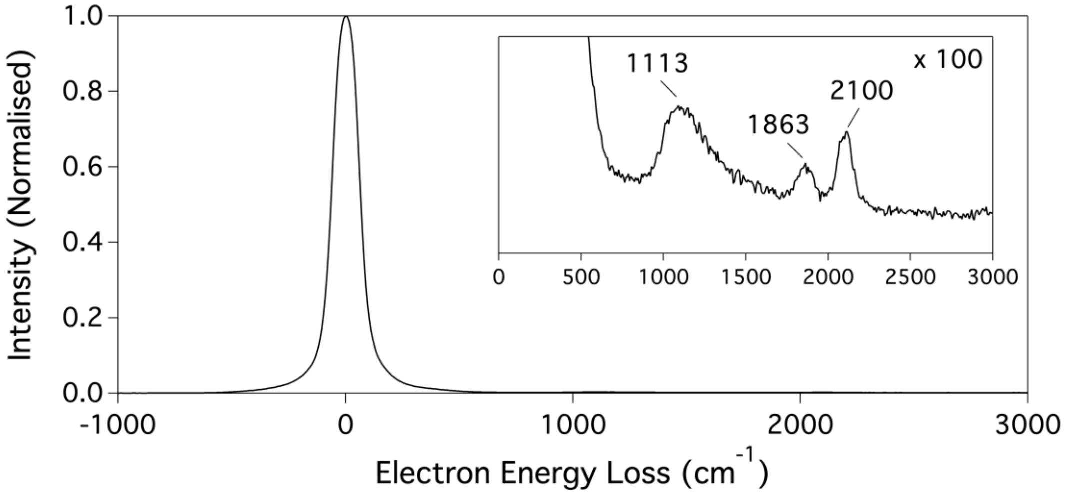

Figure S5. HREEL spectrum of Pt(111) after exposure to 10^{-8} mbar CO for one hour.

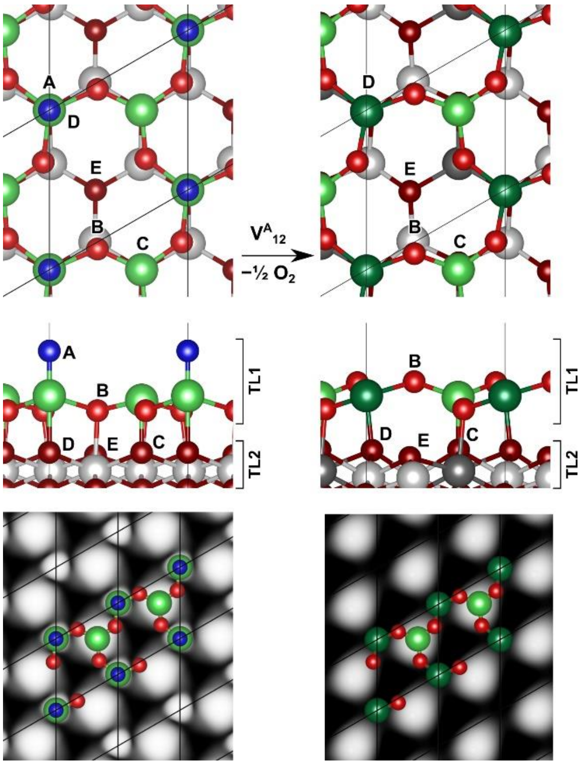

Figure S6. Simulated empty states image of the Ce=O-terminated CeO_2(111)-(√3 × √3)R30° reconstructed surface (left panel) and of the V^{A}_{12} vacancy (Table S3) structure (right panel). Ce^{4+} and Ce^{3+} ions are shown in light and dark green, respectively.

It is known that in empty states images (positive bias) of reduced ceria surfaces, Ce^{4+} ions are responsible for the contrast whereas Ce^{3+} ions are hardly visible, and therefore,`vacancies´ or dark/attenuated spots in the empty states image of the Ce=O-terminated CeO_2(111)-(√3 × √3)R30° reconstructed surface are related to the presence of Ce^{3+} ions.

Inspection of Figure 2 in the main text reveals that each surface oxygen vacancy has a vacancy or dark spot just below in the empty states image, as expected from the V^{A}_{12} configuration for which one of the excess electrons localizes right below the missing O in the outermost Ce plane (TL1), and the other one in the second cationic plane (TL2) (see Figure S6).

Moreover, we note that there are also dark spots in the empty states images at Ce positions that are not located just below the missing O sites (cf. large dark patches in Figure 2,

Figure S7) that also correspond to Ce^{3+} ions in TL1. The existence of *subsurface* vacancy structures similar or somewhat less stable than V^{A}_{12} such as V^{B}_{12}, V^{D}_{12}, V^{C}_{12} and V^{E}_{12} (Table S3), with one of the excess electrons localized in TL1, would explain those dark spots as well as the dark areas in the empty states STM images.

Finally, we note that *subsurface* vacancy structures such as V^{E}_{22} (Table S3), have their two Ce^{3+} ions in TL2, which is not expected to significantly affect the empty states STM images.

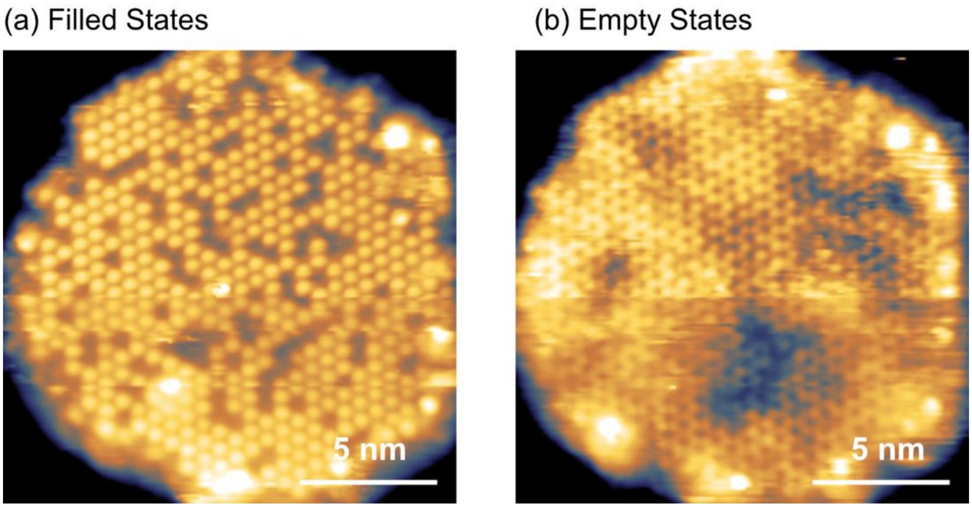

Figure S7. Atomically-resolved dual mode STM images of the top of a $CeO_2(111)$-($\sqrt{3} \times \sqrt{3}$)R30° film showing (a) Filled states, (b) Empty states.

References

1. Kresse, G.; Furthmüller, J. *Phys. Rev. B* **1996**, 54 (16), 11169–11186.
2. Kresse, G.; Joubert, D. *Phys. Rev. B* **1999**, 59 (3), 1758–1775.
3. Dudarev, S. L.; Botton, G. A.; Savrasov, S. Y.; Humphreys, C. J.; Sutton, a. P. *Phys. Rev. B* **1998**, 57 (3), 1505–1509.
4. Cococcioni, M.; de Gironcoli, S. *Phys. Rev. B* **2005**, 71 (3), 035105.
5. Fabris, S.; Vicario, G.; Balducci, G.; de Gironcoli, S.; Baroni, S. *J. Phys. Chem. B* **2005**, 109 (48), 22860–22867.
6. Perdew, J. P.; Burke, K.; Ernzerhof, M. *Phys. Rev. Lett.* **1996**, 77 (18), 3865–3868.
7. Murgida, G. E.; Ganduglia-Pirovano, M. V. *Phys. Rev. Lett.* **2013**, 110 (24), 246101.
8. Monkhorst, H. J.; Pack, J. D. *Phys. Rev. B* **1976**, 13 (12), 5188–5192.
9. J. Tersoff, D. R. Hamann, *Phys. Rev. B* **1985**, 31, 805–813.
10. Duchon J.; Dvorák, F.; Aulická, M.; Stetsovych, V.; Vorokhta, M.; Mazur, D.; Veltruská, K.; Skála, T.; Mysliveček, J.; Matolínová, I.; Matolín, V. *J. Phys. Chem. C* **2014**, 118, 357−36.
11. Olbrich, R.; Murgida, G. E.; Ferrari, V.; Barth, C.; Llois, A. M.; Reichling, M.; Ganduglia-Pirovano, M. V. *J. Phys. Chem. C* **2017**, 121, 6844−6851.
12. Luches, P.; Pagliuca, F.; Valeri, S.; Illas, F.; Preda, G.; Pacchioni, G. *J. Phys. Chem. C*, **2012**, 116, 1122-1132
13. Grinter, D. C.; Ithnin, R.; Pang, C. L.; Thornton, G. *J. Phys. Chem. C* **2010**, 114, 17036−17041.
14. Torbrügge, S.; Reichling, M. *Phys. Rev. Lett.* **2007**, 99, 56101−56105.
15. Höcker, J.; Duchon, T.; Veltruská, K.; Matolín, V.; Falta, J.; Senanayake, S. D.; Flege, J. I. *J. Phys. Chem. C* **2016**, 120, 4895−4901.
16. Wilkens, H.; Schuckmann, O.; Oelke, R.; Gevers, S.; Schaefer, A.; Bäumer, M.; Zoellner, M. H.; Schroeder, T.; Wollschläger, J. *Appl. Phys. Lett.* **2013**, 102, 111602−111605.
17. Wilkens, H.; Schuckmann, O.; Oelke, R.; Gevers, S.; Reichling, M.; S.; Schaefer, A.; Bäumer, M.; Zoellner, M. H.; Niu, G.; Schroeder, T.; Wollschläger, J. *Phys. Chem. Chem. Phys.* **2013**, 15, 18589−18599.
18. Höcker, J.; Mentes, T. O.; Sala, A.; Locatelli, A.; Schmidt, T.; Falta, J.; Senanayake, S. D.; Flege, J. I. *Adv Mater. Interf.* **2015**, 2, 1500314.
19. Luches, P.; Pagliuca, F.; Valeri, S. *Phys. Chem. Chem. Phys.* **2014**, 16, 18848-18857.

20. Lustemberg, P. G.; Pan, Y.; Shaw, B. J.; Grinter, D. C.; Pang, C. L.; Thornton, G.; Pérez, R.; Ganduglia-Pirovano, M. V.; Nilius, N. *Phys. Rev. Lett.* **2016**, 116, 236101.
21. Eck, S.; Castellarin-Cudia, C.; Surnev, S.; Ramsay, M.G.; Netzer, F.P. *Surf. Sci.* **2002**, 520, 173-185.
22. Schilling, C.; Hofmann, A.; Hess, C.; Ganduglia-Pirovano, M. V. *J. Phys. Chem. C* **2017**, 121, 20834−20849.
